# RND type efflux pump system MexAB-OprM of *pseudomonas aeruginosa* selects bacterial languages, 3-oxo-acyl-homoserine lactones, for cell-to-cell communication

**DOI:** 10.1186/1471-2180-12-70

**Published:** 2012-05-10

**Authors:** Shu Minagawa, Hiroyuki Inami, Tomohisa Kato, Shinji Sawada, Tatsuya Yasuki, Shinichi Miyairi, Manabu Horikawa, Jun Okuda, Naomasa Gotoh

**Affiliations:** 1 Department of Microbiology and Infection Control Science, Kyoto Pharmaceutical University, Yamashina, Kyoto, 607-8414, Japan; 2 Laboratory of Organic Chemistry, School of Pharmacy, Nihon University, Funahashi, Chiba, 274-8555, Japan; 3 Suntory Foundation for Life Science, Bioorganic Research Institute, Mishima, Osaka, 618-8503, Japan

## Abstract

**Background:**

Bacteria release a wide variety of small molecules including cell**-**to**-**cell signaling compounds. Gram-negative bacteria use a variety of self-produced autoinducers such as acylated homoserine lactones (acyl**-**HSLs) as signal compounds for quorum sensing (QS) within and between bacterial species. QS plays a significant role in the pathogenesis of infectious diseases and in beneficial symbiosis by responding to acyl**-**HSLs in *Pseudomonas aeruginosa*. It is considered that the selection of bacterial languages is necessary to regulate gene expression and thus it leads to the regulation of virulence and provides a growth advantage in several environments. In this study, we hypothesized that RND-type efflux pump system MexAB**-**OprM of *P. aeruginosa* might function in the selection of acyl**-**HSLs, and we provide evidence to support this hypothesis.

**Results:**

Loss of MexAB**-**OprM due to deletion of *mexB* caused increases in QS responses, as shown by the expression of *gfp* located downstream of the *lasB* promoter and LasB elastase activity, which is regulated by a LasR**-**3**-**oxo**-**C12**-**HSL complex. Either complementation with a plasmid containing wild**-**type *mexB* or the addition of a LasR**-**specific inhibitor, patulin, repressed these high responses to 3**-**oxo**-**acyl**-**HSLs. Furthermore, it was shown that the acyl**-**HSLs**-**dependent response of *P. aeruginosa* was affected by the inhibition of MexB transport activity and the *mexB* mutant. The *P. aeruginosa* MexAB**-**OprM deletion mutant showed a strong QS response to 3**-**oxo**-**C10**-**HSL produced by *Vibrio anguillarum* in a bacterial cross**-**talk experiment.

**Conclusion:**

This work demonstrated that MexAB**-**OprM does not control the binding of LasR to 3-oxo-Cn-HSLs but rather accessibility of non-cognate acyl-HSLs to LasR in *P. aeruginosa*. MexAB**-**OprM not only influences multidrug resistance, but also selects acyl**-**HSLs and regulates QS in *P. aeruginosa*. The results demonstrate a new QS regulation mechanism via the efflux system MexAB**-**OprM in *P. aeruginosa*.

## Background

Gram**-**negative bacteria use a variety of self**-**produced autoinducers such as acylated homoserine lactones as a language for quorum sensing (QS) within and between bacterial species. Several bacterial species synthesize specific acylated homoserine lactones (acyl**-**HSLs) by means of a LuxI**-**type enzyme, and respond to cognate acyl**-**HSL by using a LuxR-type intracellular receptor [1,2]. It is considered that the selection of bacterial languages is necessary to regulate gene expression and thus it leads to a growth advantage in several environments.

The opportunistic bacterium *P. aeruginosa* is widespread in various environments and utilizes two acyl-HSL signaling molecules, N**-**(3**-**oxododecanoyl)**-**L**-**homoserine lactone (3**-**oxo**-**C12**-**HSL), and N**-**butanoyl**-**L**-**homoserine lactone (C4**-**HSL), and two receptor proteins, LasR and RhlR, respectively [3]. 3-oxo-C12-HSL binds to LasR and activates LasR function. The 3-oxo-C12-HSL-LasR complex regulates many genes, including the *rhl* system [4-6]. Furthermore, *P. aeruginosa* uses a third signal, *Pseudomonas* quinolone signal (PQS) and the PqsR receptor protein [7]. Expression of many virulence factors is regulated by QS in *P. aeruginosa*[4-6,8,9]. Accordingly, a specific response to an autoinducer is important to determine the virulence of *P. aeruginosa*.

Analysis of the crystal structures of the N**-**terminal half of the *P. aeruginosa* full-length LasR or the crystal structure of *A. tumefaciences* full**-**length TraR, which is a homolog of *P. aeruginosa* LasR, in a complex with its cognate autoinducer has been performed [6,10]. These structural analyses indicated that the N-terminal half of the full length LuxR-type protein includes the dimerization domain and the acyl-HSL binding domain [6,10]. These reports indicated that the ligand binds to the N-terminal half of the full-length LuxR-type protein at an enclosed cavity far from the N-terminal dimerization region. It has been suggested that the acyl side**-**chain length of acyl**-**HSLs is not the main factor that determines the specificity of receptor protein binding [6,10]. It is considered that the binding model for the acyl-HSL-LuxR transcriptional protein family is common among Gram-negative bacteria [6,10]. However, it was shown that the responses to acyl-HSLs in *P. aeruginosa* are specific [4,11]. We hypothesize that there is an unidentified signal selection mechanism for the selection of acyl-HSLs according to the binding affinity of LasR in *P. aeruginosa*.

Resistance-nodulation-division (RND)-type efflux pumps are one type of antibiotic efflux system. RND-type efflux pumps are commonly found in gram-negative bacteria. RND family transporters catalyze the active efflux of many antibiotics and chemotherapeutic agents. They consist of an inner-membrane component belonging to the RND superfamily of secondary transporters, a channel-forming outer membrane factor (OMF), and a periplasmic membrane fusion protein (MFP) to achieve the direct extrusion of substrates across the two membranes of gram-negative bacteria [12].

The major *P. aeruginosa* RND-type efflux pump, MexAB**-**OprM provides the bacterium natural resistance to a broad spectrum of antibiotics and is not just for antimicrobial resistance [12]. On the other hand, it was reported that MexAB**-**OprM participates in the efflux of acyl-HSLs from *P. aeruginosa*[13,14]*.* These reports indicated that *P. aeruginosa* cells are not freely permeable to 3**-**oxo**-**C12**-**HSL in contrast to C4**-**HSL. Instead, it was shown that MexAB**-**OprM is involved in the active efflux of 3**-**oxo**-**C12**-**HSL [13,14]. Furthermore, a MexAB**-**OprM deletion mutant has a decreased capacity to invade or transmigrate across MDCK cells [15]. It was considered that QS**-**regulated virulence factors are affected by the MexAB**-**OprM efflux pump activity.

In this study, we hypothesized that MexAB**-**OprM of *P. aeruginosa* might function in the selection of acyl**-**HSLs, and we provide evidence to support this hypothesis. To examine the QS responses to several exogenous acyl**-**HSLs in *P. aeruginosa*, herein we focused on the *las* system because this system controls the *rhl* system and the PQS system hierarchically in *P. aeruginosa*[2,5,7]. These studies indicate that MexAB-OprM prevents the access of exogenous 3**-**oxo**-**acyl**-**HSLs to LasR, and thus LasR binds specifically to 3**-**oxo**-**C12**-**HSL. The results demonstrate a new QS regulation mechanism via the efflux system MexAB**-**OprM in *P. aeruginosa*.

## Results

### MexAB-OprM selects acyl-HSLs and regulates quorum sensing

**Figure 1:**
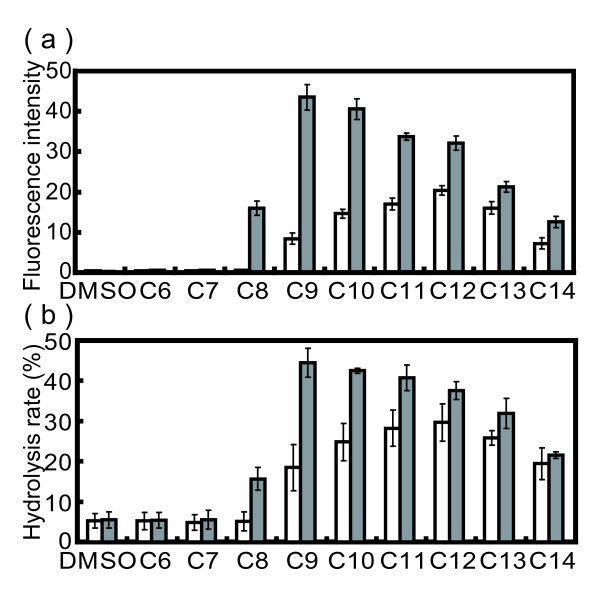
**Acyl chain length of N-(3-oxoacyl)-L-homoserine lactones has an effect on the regulation of*lasB*expression in the*mexB*deletion strain.** (**a**) Individual cultures of KG7403 (*ΔlasI ΔrhlI* P*lasB*-*gfp*) and KG703 (*ΔlasI ΔrhlIΔmexB* P*lasB*-*gfp*) were grown in LB medium containing 5 μM 3-oxo-Cn-HSL, respectively. Transcription of *lasB* was determined by measurement of the fluorescence intensity (arbitrary units) depending on the amount of green-fluorescence protein (GFP) derived from P*lasB*-*gfp* (emission at 490 nm**;** excitation at 510 nm). (**b**) Individual culture supernatants of KG7403 (*ΔlasI ΔrhlI* P*lasB*-*gfp*) and KG7503 (*ΔlasI ΔrhlIΔmexB* P*lasB*-*gfp*) grown in LB medium containing 5 μM 3-oxo-Cn-HSL, respectively, were assayed for LasB elastase activity. LasB activity was measured as the rate of hydrolysis of FRET-AGLA by the LasB protein. Hydrolysis rates were determined by measurement of fluorescence intensity depending on the N-methylanthranilyl derivative derived from an elastase substrate; emission at 355 nm and excitation at 460 nm. Open bars, KG7403; closed bars, KG7503. The data represent mean values of three independent experiments. Error bars represent the standard errors of the means.

To determine whether or not the expression of the QS regulatory pathway in *P. aeruginosa* is influenced by exogenous acyl-HSLs substituted with 3-oxo-acyl groups with carbon numbers of 6 to 14, *lasB* transcription was measured by using a *lasB* promoter-*gfp* reporter system. As a result, *lasB* transcription was most strongly induced by 3-oxo-C12-HSL, which is a cognate acyl-HSL in *P. aeruginosa* KG7403 (*ΔlasI ΔrhlI plasB-gfp*) (Figure 1a). Moreover, transcription of *lasB* resulted in a response to exogenous acyl-HSLs substituted with 3-oxo-acyl-groups with 8–14 carbons. On the other hand, we analyzed the effect of C4-HSL on *lasB* expression. The results indicated that C4-HSL was not involved in *lasB* expression (data not shown). It was previously shown that C4-HSL did not affect LasR activation [5]. Our data agree with results in this report. These results indicate that regulation of QS in *P. aeruginosa* is affected by 3-oxo-Cn-HSLs besides 3-oxo-C12-HSL.

To determine whether or not the QS system in *P. aeruginosa* is regulated by MexAB-OprM, *lasB* transcription was measured by using KG7503 (*ΔlasI ΔrhlIΔmexB plasB-gfp*). *lasB* transcription was induced to different levels by 3-oxo-Cn-HSLs with acyl chain lengths of C8 to 14 in KG7503, and compared to the results for the QS-negative mutant (Figure 1a). In this case, 3-oxo-C9-HSL (5.2-fold) and 3-oxo-C10-HSL (2.8-fold) in particular were found to induce *lasB* expression. LasB elastase activity was measured by using a FRET-AGLA-based elastase assay, similar to the *lasB*-*gfp* reporter assay (Figure 1b). The results showed that LasB activity agreed with the *lasB* transcription results (Figure 1).

The results indicate that the responses to 3-oxo-Cn-HSLs were affected by deletion of the MexAB-OprM efflux pump, and MexAB-OprM played a role in the efflux of 3-oxo-Cn-HSLs with acyl chain lengths of C8 to 14 including 3-oxo-C12-HSL. However, the QS response was more strongly induced by 3-oxo-C9-HSL or 3-oxo-C10-HSL than by 3-oxo-C12-HSL in the MexAB-OprM deletion mutant. These results suggest that the rates of 3-oxo-C9-HSL and 3-oxo-C10-HSL uptake were higher than that of 3-oxo-C12-HSL uptake, or that 3-oxo-C9-HSL and 3-oxo-C10-HSL clearance rates may be lower than that of 3-oxo-C12-HSL. Alternatively, the binding affinities of 3-oxo-C9-HSL and 3-oxo-C10-HSL to LasR were stronger than that of 3-oxo-C12-HSL.

### MexAB-OprM plays a role in the efflux of 3-oxo-cn-HSLs in *P. aeruginosa*

**Figure 2:**
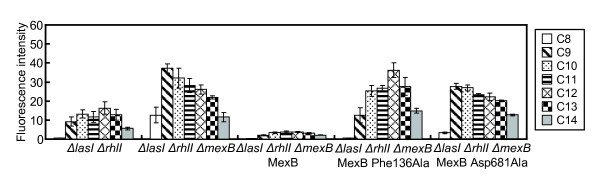
**Mutation in the predicted porter domain of MexB affected the selective efflux of aycl-HSLs by MexAB-OprM.***P. aeruginosa* strains were grown in LB medium with acyl-HSLs**,** and *lasB* expression analyses were performed as described in Materials and Methods. Promoter activities are expressed in fluorescence intensities (arbitrary units) depending on amounts of green-fluorescence protein (GFP) derived from P*lasB*-*gfp* at emission (490 nm; excitation, 510 nm). The following MexB mutant strains were used: KG7403, KG7503, KG7503 carrying pKTA113 (wild-type MexB), pYT57 (MexB Phe136Ala), and pYT81 (MexB Asp681Ala). The data represent mean values of three independent experiments. Error bars represent the standard errors of the means.

It is known that MexAB-OprM is expressed constitutively in wild-type *P. aeruginosa*, and MexAB-OprM exports a variety of substrates [10,16]. *P. aeruginosa* MexB has high sequence similarity (69.8% amino acid identity and 83.2% similarity) with *E. coli* AcrB. The crystal structure of AcrB has been solved [17,18]. The efficiency of substrate binding most likely depends on the volume and the side-chain arrangements of the binding pocket [17,18]. We attempted to model the MexB three-dimensional structure using the crystal structure of AcrB from *E. coli* by S. Murakami et al. [17,18]. Phenylalanine residues in the pore domain and hydrophobic amino acid residues in the vestibule domain were assumed to play important roles in the transport of substrates. To analyze whether a mutation in the pore domain (Phe136Ala) and a mutation in the vestibule domain (Asp681Ala) of MexB are important for extrusion of substrates, the plasmid-borne *mexB* gene was mutagenized to obtain these single-amino-acid substitutions (Figure 2). Western immunoblotting subsequently confirmed that expression of wild-type and mutant MexBs was equivalent (data not shown). *lasB* transcription was more strongly induced by acyl-HSLs in the strain carrying the MexB Phe136Ala mutation compared to the strain carrying wild-type MexB. On the other hand, *lasB* expression in response to acyl-HSLs in the MexB Asp681Ala mutant was similar to the *lasB* expression pattern in the *mexB* deletion mutant (Figure 2). *lasB* expression was affected by the mutation of these residues at positions 136 and 681 in MexB. These results indicate that MexB is necessary to extrude acyl-HSLs.

**Figure 3:**
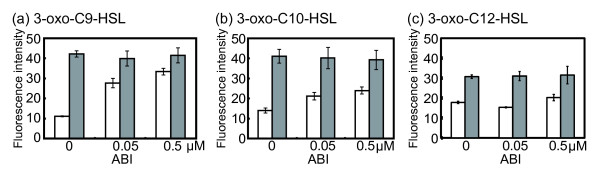
**3-oxo-Cn-HSLs are selected by MexAB-OprM in*P. aeruginosa*.** Individual cultures of KG7403 (*ΔlasI ΔrhlI* P*lasB*-*gfp*) and KG7503 (*ΔlasI ΔrhlIΔmexB* P*lasB*-*gfp*) were grown in LB medium with 5 μM 3-oxo-C9-HSL (**a**), 3-oxo-C10-HSL (**b**), or 3-oxo-C12-HSL (**c**), respectively. Transcription of *lasB* was determined by measurement of the fluorescence intensity (arbitrary units) depending on the amount of green-fluorescence protein (GFP) derived from P*lasB*-*gfp*; emission at 490 nm and excitation at 510 nm. MexAB-OprM efflux activity was inhibited by 0, 0.05 or 0.5 μM ABI. Open bars, KG7403; closed bars, KG7503. The data represent mean values of three independent experiments. Error bars represent the standard errors of the means.

Furthermore, the responses to acyl-HSLs were analyzed in the presence of the MexAB-OprM specific inhibitor ABI (Figure 3). This analysis was carried out by using a *lasB* promoter**-***gfp* reporter system with the *P. aeruginosa* cognate signal, 3-oxo-C12-HSL, and signals that strongly induce *lasB* expression, 3-oxo-C9-HSL and 3-oxo-C10-HSL. The results showed that the response to 3-oxo-C9-HSL or 3-oxo-C10-HSL was increased by ABI in a concentration-dependent manner in the MexAB-OprM activated strain (Figure 3a and b). However, the response to 3-oxo-C12-HSL was affected only by the addition of 0.5 μM ABI (Figure 3c). The analysis of MexAB-OprM inhibition by ABI showed that the effect of ABI concentration on the response of 3-oxo-C12-HSL was lower than that of 3-oxo-C9-HSL or 3-oxo-C10-HSL (Figure 3). In contrast, the response was unaffected at a range of experimental concentrations of ABI in the QS**-**negative *mexB* deletion strain (Figure 3). These results indicate that MexAB-OprM extrudes 3-oxo-Cn-HSLs from inside the cell, and that there are differences in the rates of efflux of 3-oxo-acyl-HSLs via MexAB-OprM.

The transcript levels of the *mexB* genes in the presence or absence of 3-oxo-C12-HSL were measured by semi-quantitative real-time reverse transcription**-**PCR (qRT-PCR). 3-oxo-C12-HSL had no effect on the *mexB* expression level in the QS-negative strain (data not shown), so MexAB-OprM is regulated through a QS-independent mechanism.

### LasR is activated by accumulated intracellular noncognate acyl-HSLs

**Figure 4:**
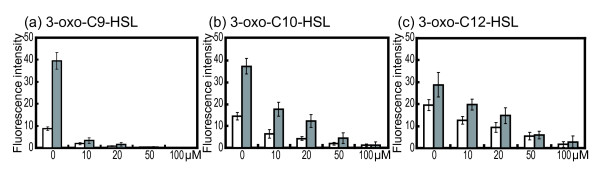
**3-oxo-Cn-HSLs bind directly to LasR and the complexes are able to trigger*lasB*expression.** Individual cultures of KG7403 (*ΔlasI ΔrhlI* P*lasB*-*gfp*) and KG7503 (*ΔlasI ΔrhlIΔmexB* P*lasB*-*gfp*) were grown in LB medium with 5 μM 3-oxo-C9-HSL (**a**), 3-oxo-C10-HSL (**b**), or 3-oxo-C12-HSL (**c**) with 0, 10, 20, 50, or 100 μM patulin, respectively. Transcription of *lasB* was determined by measuring the fluorescence intensity (arbitrary units) depending on the amounts of green-fluorescence protein (GFP) derived from P*lasB*-*gfp*; emission at 490 nm and excitation at 510 nm. Open bars, KG7403; closed bars, KG7503. The data represent mean values of three independent experiments. Error bars represent the standard errors of the means.

It is known that the overexpressed QS regulator TraR responds to a variety of autoinducers in *Agrobacterium tumefaciens*[10,19]. Thus it appears that overexpressed regulatory proteins mis-respond to acyl**-**HSL signals. In the *mexABoprM* mutant, accumulated acyl-HSLs may be bound to LasR. To verify whether or not LasR responds to 3-oxo-Cn-HSLs (C8-C14) in the MexAB-OprM deletion mutant, transcription of *lasB* in response to 3-oxo-C9-HSL, 3-oxo-C10-HSL or 3-oxo-C12-HSL was analyzed by using the LasR inhibitor, patulin (Figure 4). *lasB* induction by 3-oxo-C9-HSL, 3**-**oxo**-**C10**-**HSL or 3-oxo-C12-HSL decreased with or without MexAB-OprM in a patulin**-**concentration-dependent manner (Figure 4). These results indicate that 3-oxo-Cn-HSLs were able to bind to LasR and modulate its activity. Furthermore, the *lasB* induction by 3-oxo-C9-HSL with the addition of 10 μM patulin decreased to 10% of the level in the absence of patulin (Figure 4a). The addition of 3-oxo-C10-HSL or 3-oxo-C12-HSL with patulin decreased the *lasB* expression levels to 50% and 60%, respectively (Figure 4b and c). These data indicate that the order of LasR-binding affinity for 3-oxo-Cn-HSLs is: 3-oxo-C12-HSL > 3-oxo-C10-HSL > 3-oxo-C9-HSL. These results suggest that acyl-HSL entry into the cell is likely to be passive and acyl-HSLs were extruded by MexAB-OprM. As a result of the accumulation of these acyl-HSLs in the MexAB-OprM mutant, a non-natural response was induced.

### Selection of a bacterial language by MexAB-OprM in bacterial communication

**Figure 5:**
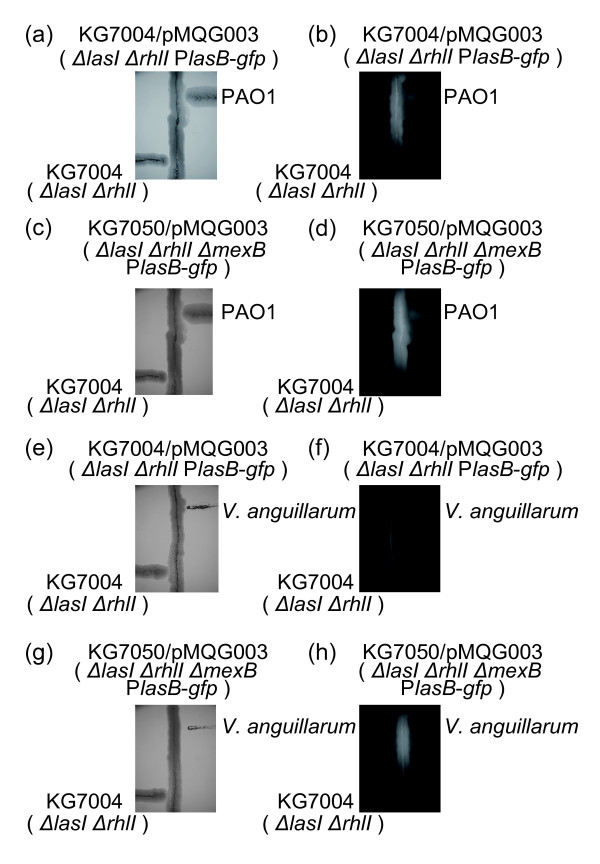
**Role of MexAB-OprM in cross-talk between*P. aeruginosa*and*V. anguilarum.*** The two monitor strains, KG7004 (*ΔlasI ΔrhlI*) and KG7050 (*ΔlasI ΔrhlIΔmexB*) harboring the *lasB* promoter-*gfp* plasmid (pMQG003) were used. *P. aeruginosa* PAO1(**a**, **b**, **c** and **d**) or *V. anguilarum* (**e**, **f**, **g** and **h**) and *P. aeruginosa* KG7004 (bottom), were cross-streaked on a LB agar plate against a monitor strain (center). Following 24 h incubation at 30°C, growth of the strains was observed under a stereomicroscope (**a**, **c**, **e** and **g**), and then production of GFP by the monitor strains was visualized by excitation of the plates with blue light (**b**, **d**, **f** and **h**).

As we have shown here, *P. aeruginosa* responds to several 3-oxo-Cn-HSLs in vitro. However, it was not known whether this in vitro response to 3-oxo-Cn-HSLs was equivalent to a response to 3-oxo-Cn-HSLs in a natural environment. When grown in close proximity to the *P. aeruginosa* wild-type strain on LB plates, KG7004 (*ΔlasIΔrhlI*) carrying pMQG003 (*lasB* promoter-*gfp*) exhibited bright-green fluorescence, but the *P. aeruginosa* reporter strain near the QS-negative strain, KG7004 (*ΔlasIΔrhlI*), did not show GFP fluorescence (Figure 5). These results clearly demonstrated that physiological concentrations of AHLs derived from PAO1 were detectable as GFP fluorescence in KG7004 (*ΔlasIΔrhlI*) carrying pMQG003 (*lasB* promoter-*gfp*) (Figure 5). To examine the effect of MexAB-OprM on heterogeneous bacterial communication, *P. aeruginosa* was co-cultivated with *C. violaceumP. chlororaphisP. agglomeransP. fluorescens* or *V. anguillarum* (Figure 5 and Additional file 1: Figure S1). These bacteria are known to produce cognate acyl-HSLs [20-23]. It was shown that *lasB* expression by *P. aeruginosaΔmexB* was only strongly induced during co-cultivation with *V. anguillarum* (Figure 5 and Additional file 1: Figure S1). 3-oxo-C10-HSL production by *V. anguillarum* was confirmed by TLC assays using *Chromobacterium violaceum* VIR07, in agreement with a previous report ( Additional file 2: Figure S2) [22].

These results indicated cross-talk via 3-oxo-C10-HSL between *P. aeruginosa* and *V. anguillarum* with the *P. aeruginosa mexAB*-*oprM* deletion strain. The transport of acyl-HSLs by MexAB-OprM plays a role in regulation of cell-cell communication.

## Discussion

The bacterial communication QS system plays many roles in the regulation of growth, biofilms, virulence and pathogenesis. Gram-negative bacteria produce specific acyl-HSLs, and then respond to specific signals. In *P. aeruginosa*, QS regulates many genes in response to the cognate 3-oxo-C12-HSL. The selection of cognate acyl-HSLs from among several autoinducers is a bacterial adaptation to environmental conditions. We showed that *P. aeruginosa* QS responds to exogenous acyl-HSLs substituted with 3-oxo-acyl-groups with between 8 and 14 carbons (Figure 1). *P. aeruginosa* LasR responds to a variety of AHLs with varying acyl chain lengths and activated LasR regulates the expression of many genes. An *A. tumefaciens* or *C. violaceum* QS reporter strain, which recognizes a broad range of acyl-HSLs, has been utilized to detect acyl-HSLs in many studies [19,22,23]. Based on these reports, it was suggested that TraR family proteins including LasR respond to several acyl-HSLs in un-natural conditions, in which the TraR family proteins are overexpressed.

The response to and specificity of the cognate bacterial language were analyzed in *P. aeruginosa* and *B. cepacia*[11]. These results suggest that bacteria have a selection mechanism for acyl-HSLs besides recognition of acyl-HSLs by the TraR family. In fact, LasR was activated by 3-oxo-C9-HSL or 3-oxo-C10-HSL in the same way as 3-oxo-C12-HSL in the *P. aeruginosa mexB* deletion mutant (Figures. 1 and 2). Furthermore, the responses to acyl-HSLs were analyzed using a site-directed MexB mutant (Figure 2). These data indicated that *lasB* expression was affected by the substitutions Phe136Ala or Asp681Ala in MexB (Figure 2). In particular, the MexB Phe136Ala mutation affected the response to acyl-HSLs similar to that of the *mexB* deletion mutant (Figure 2). This result suggested that Phe136 in MexB played an important role in substrate extrusion by MexB. On the other hand, *lasB* expression increased in the MexB Asp681Ala mutant compared with wild-type MexB. This result suggested that the MexBAsp681Ala mutation induced the extrusion activity of MexB. Recently, the crystal structure of MexB from *P. aeruginosa* was resolved and suggests a mechanism for substrate transport by MexB [24]. The residues at positions 136 in MexB are located in between the PN1 subdomain and the PN2 subdomain [24]. The residues at positions 681 in MexB are located in the PC2 subdomain [24]. The PC2 domain plays an important role in the formation of the entrance channel [24]. These data support the suggestion that Phe136 in MexB plays an important role in substrate extrusion by MexB.

MexAB-OprM inhibition by ABI showed that the LasR activation by 3-oxo-C9-HSL or 3-oxo-C10-HSL was similar to that in the *mexB* deletion mutant (Figures 1 and 3). The effect of ABI concentration on the response to 3-oxo-C12-HSL was lower than that of 3-oxo-C9-HSL or 3-oxo-C10-HSL (Figure 3). These data suggest that the difference in the efflux ratio of 3-oxo-acyl-HSLs via MexAB-OprM may be due to differences in the acyl-side chain lengths; these differences in the efflux ratio were important in the response to the cognate 3-oxo-C12-HSL in *P. aeruginosa*. However, we have to consider the degradation of acyl-HSLs by QS quenching lactonases or acylases, as well as LasR acyl-HSL binding activity in the acyl-HSLs response in *P. aeruginosa*. Previous studies showed that the substrate specificity of QS quenching enzymes was broad [25,26]. In addition, we showed the LasR responds to several acyl-HSLs by using the patulin competition assay (Figure 4). These results support the hypothesis that *P. aeruginosa* needs to use the acyl-HSLs selection system of MexAB-OprM in order to respond to cognate acyl-HSLs in mixed bacterial culture conditions. Furthermore, it is known that the concentrations of acyl-HSLs are high at high cell densities and LasR binds its specific acyl-HSL to activate the LasR regulon [4]. It was also suggested that MexAB-OprM regulates the concentration of acyl-HSLs in the cell via acyl-HSLs extrusion. The regulation of acyl-HSLs concentration via MexAB-OprM may therefore be important in the *P. aeruginosa* QS response*.*

The *P. aeruginosa mexABoprM* deletion mutant responded to 3-oxo-C10-HSL produced by *V. anguillarum* during *P. aeruginosaV. anguillarum* co-cultivation (Figure 5). These results indicate that intracellular acyl-HSLs exported by MexAB-OprM regulated QS in *P. aeruginosa*. It has also been reported that the RND-type efflux pump BpeAB-OprB in *B. pseudomallei* is closely involved in bacterial communication [27,28]. These findings suggest that RND-type efflux pumps have a common ability for several acyl-HSL efflux systems. This selection mechanism may result in improved survival in mixed culture conditions.

## Conclusions

**Figure 6:**
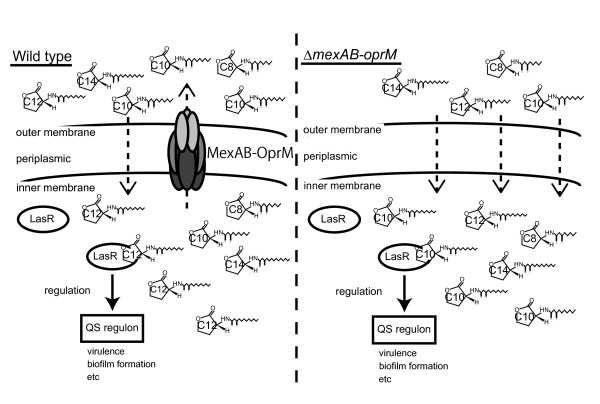
**A model for QS regulation mechanism via the RND-type efflux pump MexAB-OprM*.*** (a) MexAB-OprM extrudes 3-oxo-Cn-HSLs and controls the accessibility of non-cognate acyl-HSLs to LasR in *P. aeruginosa* QS-regulation. (b) In the *P. aeruginosa* MexAB-OprM mutant, non-cognate 3-oxo-Cn-HSLs activate LasR. Non-cognate 3-oxo-Cn-HSLs-LasR complexes induce the wrong QS regulation.

This work demonstrates that MexAB-OprM does not control the binding of LasR to 3-oxo-Cn-HSLs but rather the accessibility of non-cognate acyl-HSLs to LasR in *P. aeruginosa* (Figure 6). Furthermore, the results indicate that QS is regulated by MexAB-OprM (Figure 6). MexAB-OprM not only influences multidrug resistance, but also selects acyl-HSLs and regulates QS in *P. aeruginosa*. The results demonstrate a new QS regulation mechanism via the efflux system MexAB-OprM in *P. aeruginosa*.

## Methods

### Bacterial strains, plasmids and growth conditions

The bacterial strains and plasmids used in this study are listed in Table 1. Bacterial cells were grown in LB broth or on LB agar at 37°C or 30°C. The following antibiotics were added to media at the indicated concentrations: ampicillin, 100 μg/ml for *E. coli*; carbenicillin, 200 μg/ml for *P. aeruginosa*; tetracycline, 25 μg/ml for *E. coli*, 100 μg/ml for *P. aeruginosa*.

**Table 1:** Strains and Plasmids

**Strains/Plasmids**	**Characteristics**	**Reference**
**Strains**		
*P. aeruginosa*		
PAO1	ATCC15692	[29]
KG4509	*ΔmexB* derivative of PAO1	This study
KG7004	*ΔlasI ΔrhlI* derivative of PAO1	This study
KG7050	*ΔlasI ΔrhlIΔmexB* derivative of PAO1	This study
KG7403	*gfp* fused to the *lasB* promoter and integrated at the *attB* site of the KG7004 chromosome	This study
KG7503	*gfp* fused to the *lasB* promoter and integrated at the *attB* site of the KG7050 chromosome	This study
*E. coli*		
DH5α	F^-^, Φ80d *lacZ Δ*M15, *Δ*(*lacZYA*- *argF'*)U169, *deoR*, *recA*1, *endA*1, hsdR17(r_k_^-^ m_k_^+^), *phoA*, *supE*44, λ^-^, *thi*-1, *gyrA*96, *relA*1	[30]
S17-1	RE42-Tc: Mu-Km:: Tn7 pro res mod4	[31]
**Plasmids**		
pUC18	Ap^r^; high-copy-number cloning vector	[32]
pBR322	Ap^r^ Tc^r^; high-copy-number cloning vector	[33]
pSL1180	super-polylinker phagemid	[34]
pTO003	Gm^r^; *E. coli*-*P. aeruginosa* shuttle expression vector	[35]
pMT5059	Cb^r^; pBend2 derivative carrying multiple-cloning site and *Not* I site	[36]
pMT5071	Cm^r^; pMOB3 derivative carrying Ω-Cm instead of Cm	[37]
pAF2071	Cb^r^ Cm^r^; pKT5059 carrying 2911-bp fragment with 3′ flanking region of *rhlI* including 91-bp of *rhlI* and 2110-bp fragment with 5′ flanking region of *rhlI* Mob cassette from pMT5071 at *Not* I	This study
plasI	Cb^r^ Cm^r^; pMT5059 carrying 1.0-kb PCR fragments with 3′ and 5′ flanking regions of *lasI* and Mob cassette from pMT5071 at *Not* I	This study
pMexB	Cb^r^ Cm^r^; pMT5059 carrying 1.0-kb PCR fragments with 3′ and 5′ flanking regions of *mexB* and Mob cassette from pMT5071 at *Not* I	This study
pKTA113	Gm^r^; subcloning of full length *mexB* in *Xba* I and *Hin* dIII site of pTO003	This study
pYT57	Phe136Ala mutation of *mexB* on pKTA113	This study
pYT81	Asp681Ala mutation of *mexB* on pKTA113	This study
pGreen	Ap^r^; Enhanced GFP cassette plasmid	[38]
mini-CTX1	Tc^r^; self-profcient integration vector with tet, V-FRT-*attP*-MCS, *ori*, *int*, and *oriT*	[39]
pSG	Tc^r^; subcloning of *gfp* from pGreen	This study
pSQG003	Tc^r^; subcloning of a 598-bp *lasB* promoter region between *Hin* dIII and *Kpn* I sites of pSG	This study
pFLP2	Ap^r^; source of Flp recombinase	[40]
pME6012	Tc^r^; pVS1-p15A shuttle vector	[41]
pMQG003	Tc^r^; subcloning of a 1781-bp *lasB* promoter::*gfp* region from pSQG003 in *Bgl* II site of pME6012	This study

### Construction of knockout mutants lacking quorum sensing and efflux protein genes

The *P. aeruginosa* mutants, KG7004 and KG7050, lacking quorum sensing and efflux protein genes were constructed by allele exchange using the plasmids listed in Table 1, as described previously [30,35,42]. Construction of *P. aeruginosa* mutants in this study followed the order: PAO1 to KG7001 with plasI (for deletion of *lasI*), KG7001 to KG7004 with pAF2071 (for deletion of *rhlI*), and KG7004 to KG7050 with pMexB (for deletion of *mexB*), respectively.

### Construction of QS reporter strains

pSQG was constructed by subcloning a 700-bp *Eco*RI digested fragment derived from pGreen into the *Kpn*I site of mini-CTX1 [38,39]. A *lasB* promoter-*gfp* translational fusion was constructed by ligating a 591-bp fragment including the region encoding N-terminal ten amino acids of LasB derived from the *P. aeruginosa* PAO1 chromosome. The resulting plasmid, pSQG003, was mobilized into KG7004 and KG7050 via *E. coli* S17-1. To accomplish excision, pFLP2, encoding Flp recombinase, was introduced into the *P. aeruginosa* KG7403 and KG7503 strains containing the *lasB* promoter-*gfp* translational fusion constructs by using the high transformation method and previously described procedures [40,43].

In addition, the multicopy reporter plasmid pMQG003 was constructed. A *lasB* promoter-*gfp* translational fusion fragment from pSQG003 was cloned into pME6012 [41]. The *lasB* promoter-*gfp* translational fusion fragment was prepared by using PCR with the primers CTX1-F (5′-CGATAGATCTGCCGTCCTTGCTGAATTAGC-3′) and CTX1-R (5′-AACTAGATCTCGCTTTTGAAGCTGATGTGC-3′) containing an engineered restriction site *Bgl*II (forward and reverse). This fragment was restricted with *Bgl*II, and then ligated **to** the *Bgl*II site of pME6012.

### Construction of the plasmids expressing the wild-type and mutant *mexB* genes in *P. aeruginosa*

The stable *E. coli*–*P. aeruginosa* shuttle vector pKTA113 carrying *mexB* was constructed in three steps. The first *mexB* fragment amplified by PCR using the chromosomal DNA of *P. aeruginosa* PAO1 as a template and a pair of primers containing the engineered restriction sites *Hin*dIII (5′-ACATAAGCTTATGTCGAAGTTTTTCATTGATAGG -3′) and *Sal*I (5′- GCAATCTAGATTGCCCCTTTTCGACGGACG -3′). Next, *mexB* fragments were ligated to the multicloning site of pUC18 to yield pYT06. To obtain the MexB expression plasmid, a 3138**-**bp *Hin*dIII-*Xba*I fragment from pYT06 was ligated to the large *Hin*dIII-*Xba*I fragment of pTO003. The resulting construct containing MexB-6His under the *lac* promoter shall be referred to as pKTA113 in this paper.

To produce *mexB* mutants, the Stratagene Quickchange site-directed mutagenesis kit (Stratagene) was used according to the manufacturer’s protocol. The Phe136Ala or Asp681Ala substitution was introduced into pYT06, respectively. Then the mutated *mexB* fragments of the pYT06 mutants were subcloned into pTO003.

### Detection of *lasB* promoter activity by using GFP fluorescence intensity

Cells were grown overnight at 30°C in LB medium with shaking. Overnight cultures were subcultured into fresh LB medium at a ratio of 1:100, grown under the same conditions for three hours, and then supplemented with 5 μM 3-oxo-Cn-HSL, respectively. Following an 8 h incubation at 30°C, cells grown in LB with various acyl-HSLs were harvested by centrifugation, resuspended in phosphate-buffered saline, and then diluted with 200 μl of phosphate-buffered saline. Green fluorescence of the reporter strains was measured using a Varioskan^**TM**^ microtiter plate reader (Thermo Fisher Scientific), with an excitation wavelength of 490 nm and emission detection at 510 nm. Data are means ± standard deviations for three independent experiments. The LasR inhibitor, Patulin was obtained from Wako-Pure Chemicals Ltd. (Osaka, Japan) [8]. The MexAB-OprM specific inhibitor, ABI ([[2-({[((3R)-1-{8-{[(4-tert-butyl-1,3-thiazol-2-yl) amino]carbonyl}-4-oxo-3-[(*E*)-2-(1 *H*-tetrazol-5-yl)vinyl]-4 *H*-pyrido[1,2-a]pyrimidin-2-yl} piperidin-3-yl)oxy]carbonyl}amino)ethyl](dimethyl)ammonio]acetate, C_31_H_39_N_11_O_6_S·6H_2_O) was obtained from Daiichi Pharmaceutical Co., Ltd. (Tokyo, Japan) [44].

### Elastase assay by using FRET-AGLA

The elastase activity in a *P. aeruginosa* culture supernatant was determined by using FRET-AGLA (see Additional file 3). Cells were grown under the same conditions as the *lasB* reporter assay. Cells grown in LB with various acyl-HSLs were harvested by centrifugation, and culture supernatants were recovered and filtered (0.22 μm pore-size filter). 50 μl samples diluted 50-fold were added to tubes containing 100 μl of a FRET-AGLA solution (50 mM Tris–HCl, 200 mM NaCl (pH 7.5), 10 mM CaCl_2_, 0.4 mM FRET-AGLA). The tubes were incubated for 15 min at 30°C and then 50 μl of 1 M NaOH was added. The degradation products of FRET-AGLA produced by elastase were measured using the Varioskan^**TM**^ microtiter plate reader with an excitation wavelength of 355 nm and emission detection at 460 nm. The resolution rate of the degradation products of FRET-AGLA was determined by extrapolating the obtained fluorescence of the degradation products of FRET-AGLA on a standard curve.

### Cross-streaking experiments

The monitor strains, KG7004(pMQG003) or KG7050(pMQG003), and the respective test strains were streaked close to each other on nutrient agar plates (Nissui, Tokyo, Japan) (see 3). Following 24 h incubation at 30°C, the plates were illuminated with blue light using an SZX-FGFP filter in combination with a halogen lamp as a light source, and green fluorescence was observed under a Stereomicroscope SZX12 system (Olympus).

## Authors’ contributions

SM, and SS carried out the elastase assay and *lasB* reporter assay. HI carried out cross-streak experiments. TK constructed *lasB* promoter-*gfp* reporter strains. SM synthesized FRET-AGLA, elastase substrate. MH synthesized acyl-HSLs. JO and NG conceived of the study, and participated in its design and coordination and helped to draft the manuscript. All authors read and approved the final manuscript.

## Supplementary Material

Additional file 1 **Figure S1. Cross-streak experiment for detection of bacterial interaction via acyl-HSLs.** The two monitor strains used were KG7004 (*ΔlasI ΔrhlI*) and KG7050 (*ΔlasIΔrhlI*4 *ΔmexB*) harboring the *lasB* promoter-*gfp* plasmid (pMQG003) were used. Test strains against the monitor strains (center) were cross-streaked on LB agar plates. Following 24 h incubation at30°C, the growth of strains was observed under a stereomicroscope, and then production of GFP by the monitor strains was visualized by excitation of the plates with blue light. Click here for file

Additional file 2 **Figure S2. TLC analysis of 3-oxo-C10-HSL produced by*V. anguillarum.*** Extracted samples from *V. anguillarum* cultures were chromatographed on a C-18 RP-TLC plate, developed with methanol/water (70:30, v/v). The spots were visualized 13 by overlaying the TLC plate with *C. violaceum* VIR07. As AHL standards, Cn-HSL: 14 C6-HSL, C8-HSL and C10-HSL, 3-oxo-Cn-HSL: 3-oxo-C6-HSL, 3-oxo-C8-HSL, 15 3-oxo-C10-HSL and 3-oxo-C12-HSL were used. Click here for file

Additional file 3 **Supplemental information of Materials, Methods, Figure legend of Figure S1 and S2 and References**[1,45-49]. Click here for file
