# Supplementary material for: RND type efflux pump system MexAB-OprM of pseudomonas aeruginosa selects bacterial languages, 3-oxo-acyl-homoserine lactones, for cell-to-cell communication
Source: BMC Microbiol. 2012 May 10;12:70. doi: 10.1186/1471-2180-12-70 (PMC3460771; doi:10.1186/1471-2180-12-70)
Supplement: Additional file 1 — Figure S1. Cross-streak experiment for detection of bacterial interaction via acyl-HSLs. The two monitor strains used were KG7004 (ΔlasI ΔrhlI) and KG7050 (ΔlasIΔrhlI4 ΔmexB) harboring the lasB promoter-gfp plasmid (pMQG003) were used. Test strains against the monitor strains (center) were cross-streaked on LB agar plates. Following 24 h incubation at30°C, the growth of strains was observed under a stereomicroscope, and then production of GFP by the monitor strains was visualized by excitation of the plates with blue light. [file 1471-2180-12-70-S1.pdf]

Additional files

Materials.

(*S*)-(-)- α -Amino- γ -butyrolactone (L-homoserine lactone, HSL) HBr was obtained from Wako Pure Chemicals Ltd. (Osaka, Japan). Lithium bis(trimethylsilyl)amide (LHMDS) was obtained from Sigma-Aldrich Inc. (St. Louis, MO, USA). Methyl 3-oxohexanoate, methyl 3-oxoheptanoate, 2-heptanone, 2-octanone, 2-nonanone, 2-decanone, 2-undecanone, 2-dodecanone, 2-tridecanone, butyric acid, pentanoic acid, hexanoic acid, heptanoic acid, octanoic acid, nonanoic acid, decanoic acid, undecanoic acid, dodecanoic acid, tridecanoic acid, tetradecanoic acid, 1-ethyl-3-(3-dimethylaminopropyl)carbodiimide (EDC) HCl, *N*-*tert*-butoxycarbonyl-L-alanine (*N*-*t*-Boc-Ala), *N*-*tert*-butoxycarbonyl-L-leucine (*N*-*t*-Boc-Leu), *N*-*tert*-butoxycarbonylglycine (*N*-*t*-Boc-Gly), 4-nitrobenzylamine HCl and *N*-methylanthranilic acid were purchased from Tokyo-Kasei Kogyo Ltd. (Tokyo, Japan). L-Alanine benzyl ester (Ala-Bz) HCl and glycine benzyl ester (Gly-Bz) HCl were purchased from Bachem AG (Burbendorf, Switzerland) and Watanabe Chemicals (Hiroshima, Japan), respectively.

Preparation of N-acyl L-homoserine lactones.

N-(3-Oxohexanoyl)-HSL (3-oxo-C6-HSL), *N*-(3-oxoheptanoyl)-HSL (3-oxo-C7-HSL), *N*-(3-oxooctanoyl)-HSL (3-oxo-C8-HSL), *N*-(3-oxononanoyl)-HSL (3-oxo-C9-HSL), *N*-(3-oxodecanoyl)-HSL (3-oxo-C10-HSL), *N*-(3-oxoundecanoyl)-HSL (3-oxo-C11-HSL), *N*-(3-oxododecanoyl)-HSL (3-oxo-C12-HSL), *N*-(3-oxotridecanoyl)-HSL (3-oxo-C13-HSL), and *N*-(3-oxo-tetradecanoyl)-HSL (3-oxo-C14-HSL) were prepared in our laboratories from HSL HBr with the corresponding fatty acids by condensation using EDC HCl. 3-Oxohexanoic acid and 3-oxoheptanoic acid were prepared from the corresponding methyl esters by alkaline hydrolysis. 3-Oxooctanoic acid, 3-oxononanoic acid, 3-oxodecanoic acid, 3-oxoundecanoic acid, 3-oxododecanoic acid, 3-oxotridecanoic acid and 3-oxotetradecanoic acid were prepared from 2-heptanone, 2-octanone, 2-nonanone, 2-decanone, 2-undecanone, 2-dodecanone and 2-tridecanone by carboxylation with carbon dioxide, followed by enolation of the 2-ketoalkanes with LHMDS, respectively (1).

Synthesis of 4-nitrobenzylamide of N-(N-methylanthranilyl)-L-alaninylglycyl-L-leucyl-L-alanine (FRET-AGLA).

The synthetic elastase substrate, FRET-AGLA, was prepared in our laboratories by

the procedure described below. The amino acid sequence was cited from Nishino and Powers (2). **1**) 4-Nitrobenzylamide of Ala (Ala-NBA) HCl was prepared from *N*-*t*-Boc-Ala and 4-nitrobenzylamine HCl by a condensation reaction using EDC HCl in the presence of triethylamine (TEA), followed by hydrolysis of the *t*-Boc group in the presence of HCl in ethyl acetate (EtOAc). **2**) Leu-Ala-NBA HCl was prepared from *N*-*t*-Boc-Leu and Ala-NBA HCl by a condensation reaction using EDC HCl in the presence of TEA, followed by hydrolysis of the *t*-Boc group in the presence of HCl in a mixture of EtOAc and methanol (MeOH). **3**) Gly-Leu-Ala-NBA HCl was prepared from *N*-*t*-Boc-Gly and Leu-Ala-NBA HCl by a condensation reaction using EDC HCl in the presence of TEA, followed by hydrolysis of the *t*-Boc group as in "**2**". **4**) Then, *N*-(*N*-methylanthranilyl)-Ala (MAn-Ala), the *N*-terminal part of FRET-AGLA, was prepared separately from N-methylanthranilic acid and Ala-Bz HCl by a condensation reaction using EDC HCl in the presence of TEA, followed by removal of the benzyl group by hydrogenolysis using 10% palladium on carbon as a catalyst. **5**) Finally, FRET-AGLA was prepared from Gly-Leu-Ala-NBA HCl and MAn-Ala by a condensation reaction using EDC in the presence of TEA, and the product was purified by recrystallization from a mixture of EtOAc and MeOH. **6**) MAn-Ala-Gly, one side of the hydrolyzed product derived from FRET-AGLA by the elastase of *P. aeruginosa*, was

prepared from MAn-Ala and Gly-Bz by the condensation reaction using EDC in the presence of TEA, followed by removal of the benzyl group as in "**4**".

Physical data for FRET-AGLA.

Mass spectra (MS) and ^{1}H-nuclear magnetic resonance (NMR) spectra were recorded on JEOL GC mate and JEOL FX-600, respectively. The abbreviations used are *s*: singlet, *d*: doublet, *t*: triplet, *m*: multiplet.

MS (FAB^{+}): 598 ([M+H]$^{+}$), HR-MS(FAB^{+}): calcd for $C_{29}H_{40}N_7O_7$: 598.2989. Found: 598.2991. ^{1}H-NMR (CD_3OD) δ: 0.74 and 0.83 (each 3H, *d*. *J*=4.8 Hz, $(CH_3)_2$ of Leu), 1.42 (3H, *d*, *J*=7.2 Hz, CH_3 of Ala at *N*-terminal), 1.47 (3H, *d*, *J*=6.9 Hz, CH_3 of Ala at *C*-terminal), 1.57 (2H, *m*, CH_2 of Leu), 1.53-1.65 (1H, *m*, CH of Leu), 2.76 (3H, *s*, *N*-CH_3 of MAn), 3.74 and 3.96 (each 1H, *d*, *J*=16.9 Hz, CH_2 of Gly), 4.34 (2H, *m*, $(CH)_2$ of Ala at both terminal sides), 4.41-4.45 (1H, *m*, CH of Leu), 4.47 (2H, *s*, CH_2 of NBA), 6.59 (1H, *t*, *J*=7.9 Hz, 5-H of MAn), 6.65 (1H, *d*, *J*=7.9 Hz, 3-H of MAn), 7.31 (1H, *t*, *J*=7.9 Hz, 4-H of MAn), 7.50 (2H, *d*, *J*=7.3 Hz, 2-H and 6-H of NBA), 7.62 (1H, *d*, *J*=7.9 Hz, 6-H of MAn), 8.17 (2H, *d*, *J*=7.3 Hz, 3-H and 5-H of NBA).

Strains.

P. aeruginosa PAO1 and *Pseudomonas fluorescens* Pf-5 are laboratory stock strains.

Pseudomonas chlororaphis JCM9490 were provided by the Japan Collection of Microorganisms, RIKEN BRC, which is participating in the National BioResource Project of MEXT, Japan. *Pseudomonas agglomerans* NBRC12686 and *Vibrio anguillarum* NBRC12710 were obtained from the NITE Biological Resource Center (Kisarazu, Japan).

Methods

Cross-streaking experiments.

P. aeruginosa PAO1, *P. chlororaphis* JCM9490, *P. agglomerans* NBRC12686, *P. fluorescens* Pf-5 and *V. anguillarum* NBRC12710 were used in this experiment. It was reported that these strains produced cognate acyl-HSLs, respectively [3, 4, 5, 6]. The monitor strain, KG7004(pMQG003) or KG7050(pMQG003), and the respective test strains were streaked close to each other on nutrient agar plates (Nissui, Tokyo, Japan). Following 24 h incubation at 30℃, the plates were illuminated with blue light using an SZX-FGFP filter in combination with a halogen lamp as a light source, and green florescence was observed by using a stereomicroscope SZX12 system (Olympus).

Extraction and detection of *C. anguillarum* 3-oxo-C10-HSL.

Bacterial strains were incubated in 50 ml of nutrient broth (Nissui, Tokyo, Japan). The culture supernatant was filtered using a 0.22-mm pore-size membrane filter and then extracted with 50 ml of acidified ethyl acetate. The extracts were then evaporated to dryness and dissolved in 50 μl of ethyl acetate. Samples were analyzed by using TLC analysis. TLC analysis was carried out on C-18 reversed-phase thin layer chromatograph plates (C-18-RP-TLC plates) (Merck, Germany). AHL samples and standards were spotted on to a TLC plate and developed with 70% (v/v) methanol in water. The air-dried plate was overlaid with LB soft agar containing *C. violaceum* VIR07 and then incubated at 30℃.

Figures

Figure S1 - Cross-streak experiment for detection of bacterial interaction via acyl-HSLs.

The two monitor strains used were KG7004 (*ΔlasI ΔrhlI*) and KG7050 (*ΔlasI ΔrhlI ΔmexB*) harboring the *lasB* promoter-*gfp* plasmid (pMQG003) were used. Test strains against the monitor strains (center) were cross-streaked on LB agar plates. Following 24 h incubation at 30℃, the growth of strains was observed under a stereomicroscope, and then production of GFP by the monitor strains was visualized by excitation of the plates with blue light.

Figure S2 - TLC analysis of 3-oxo-C10-HSL produced by *V. anguillarum*.

Extracted samples from *V. anguillarum* cultures were chromatographed on a C-18 RP-TLC plate, developed with methanol/water (70:30, v/v). The spots were visualized by overlaying the TLC plate with *C. violaceum* VIR07. As AHL standards, Cn-HSL: C6-HSL, C8-HSL and C10-HSL, 3-oxo-Cn-HSL: 3-oxo-C6-HSL, 3-oxo-C8-HSL, 3-oxo-C10-HSL and 3-oxo-C12-HSL were used.

References

1. Horikawa M, Tateda K, Tuzuki E, Ishii Y, Ueda C, Takabatake T, Miyairi S, Yamaguchi K, Ishiguro M: **Synthesis of *Pseudomonas* quorum-sensing autoinducer analogs and structural entities required for induction of apoptosis in macrophages.** *Bioorg. Med. Chem. Lett.* 2006, **16:**2130-2131.
2. Nishino N, Powers JC: ***Pseudomonas aeruginosa* elastase: Development of a new substrate, inhibitors, and an affinity ligand.** *J. Biol. Chem.* 1980, **255**:3482-3486.
3. Fuqua C, Greenberg EP: **Listening in on bacteria: acyl-homoserine lactone signaling.** *Nat Rev,* 2002, **3:**685-695.
4. Chin-A-Woeng TF, van den Broek D, de Voer G, van der Drift KM, Tuinman S, Thomas-Oates JE, Lugtenberg BJ, Bloemberg GV: **Phenazine-1-carboxamide production in the biocontrol strain *Pseudomonas chlororaphis* PCL1391 is regulated by multiple factors secreted into the Growth Medium.** *Mol Plant Microbe Interact,* 2001, **14:**969-979.
5. Laue BE, Jiang Y, Chhabra SR, Jacob S, Stewart GSAB, Hardman A, Downie JA, O'Gara F, Williams P. **The biocontrol strain *Pseudomonas fluorescens* F113 produces the Rhizobium small bacteriocin, *N*-(3-hydroxy-7-cis-tetradecenoyl) homoserine lactone, via HdtS, a putative novel *N*-acylhomoserine lactone**

synthase. *Microbiol,* 2000, **146:**2469–2480.

6. Holden MT, Ram Chhabra S, de Nys R, Stead P, Bainton NJ, Hill PJ, Manefield M, Kumar N, Labatte M, England D, Rice S, Givskov M, Salmond GP, Stewart GS, Bycroft BW, Kjelleberg S, Williams P: Quorum-sensing cross talk: isolation and chemical characterization of cyclic dipeptides from Pseudomonas aeruginosa and other gram-negative bacteria. *Mol Microbiol.* 1999, **33**:1254-1266.
