# Supplementary material for: RND type efflux pump system MexAB-OprM of pseudomonas aeruginosa selects bacterial languages, 3-oxo-acyl-homoserine lactones, for cell-to-cell communication
Source: BMC Microbiol. 2012 May 10;12:70. doi: 10.1186/1471-2180-12-70 (PMC3460771; doi:10.1186/1471-2180-12-70)
Supplement: Additional file 2 — Figure S2. TLC analysis of 3-oxo-C10-HSL produced byV. anguillarum. Extracted samples from V. anguillarum cultures were chromatographed on a C-18 RP-TLC plate, developed with methanol/water (70:30, v/v). The spots were visualized 13 by overlaying the TLC plate with C. violaceum VIR07. As AHL standards, Cn-HSL: 14 C6-HSL, C8-HSL and C10-HSL, 3-oxo-Cn-HSL: 3-oxo-C6-HSL, 3-oxo-C8-HSL, 15 3-oxo-C10-HSL and 3-oxo-C12-HSL were used. [file 1471-2180-12-70-S2.pdf]

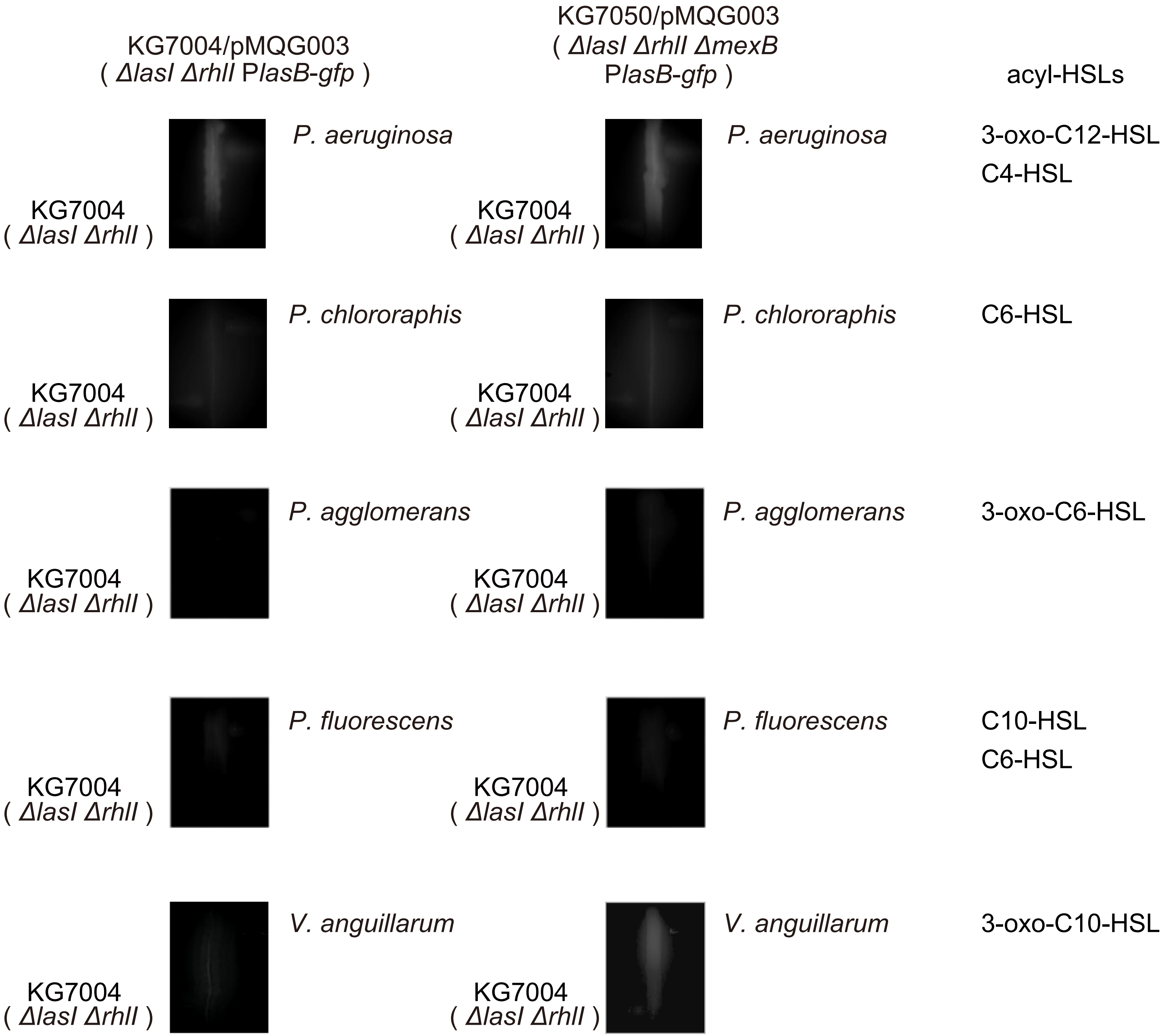

KG7004/pMQG003
(ΔlasI ΔrhlI PlasB-gfp)
KG7050/pMQG003
(ΔlasI ΔrhlI ΔmexB
PlasB-gfp)
acyl-HSLs
P. aeruginosa
KG7004
(ΔlasI ΔrhlI)
P. aeruginosa
KG7004
(ΔlasI ΔrhlI)
3-oxo-C12-HSL
C4-HSL
P. chlororaphis
KG7004
(ΔlasI ΔrhlI)
P. chlororaphis
KG7004
(ΔlasI ΔrhlI)
C6-HSL
P. agglomerans
KG7004
(ΔlasI ΔrhlI)
P. agglomerans
KG7004
(ΔlasI ΔrhlI)
3-oxo-C6-HSL
P. fluorescens
KG7004
(ΔlasI ΔrhlI)
P. fluorescens
KG7004
(ΔlasI ΔrhlI)
C10-HSL
C6-HSL
V. anguillarum
KG7004
(ΔlasI ΔrhlI)
V. anguillarum
KG7004
(ΔlasI ΔrhlI)
3-oxo-C10-HSL
