# Supplementary figures and images for: RND type efflux pump system MexAB-OprM of pseudomonas aeruginosa selects bacterial languages, 3-oxo-acyl-homoserine lactones, for cell-to-cell communication
Source: BMC Microbiol. 2012 May 10;12:70. doi: 10.1186/1471-2180-12-70 (PMC3460771; doi:10.1186/1471-2180-12-70)

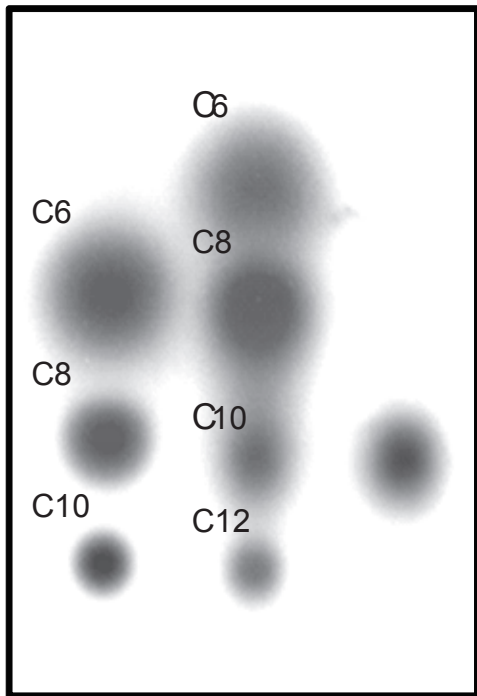

Cn-HSL 3-oxo-Cn-HSL *V. anguillarum*

Supplement: Additional file 3 — Supplemental information of Materials, Methods, Figure legend of Figure S1 and S2 and References[1,45-49]. [file 1471-2180-12-70-S3.pdf]
